# Behavioural synchronization in a multilevel society of feral horses

**DOI:** 10.1371/journal.pone.0258944

**Published:** 2021-10-26

**Authors:** Tamao Maeda, Cédric Sueur, Satoshi Hirata, Shinya Yamamoto

**Affiliations:** 1 Wildlife Research Centre, Kyoto University, Kyoto, Japan; 2 Institut Pluridisciplinaire Hubert Curien, Université de Strasbourg, CNRS, Strasbourg, France; 3 Institut Universitaire de France, Paris, France; 4 Institute for Advanced Study, Kyoto University, Kyoto, Japan; Institut de Recherche pour le Developpement, FRANCE

## Abstract

Behavioural synchrony among individuals is essential for group-living organisms. The functioning of synchronization in a multilevel society, which is a nested assemblage of multiple social levels between many individuals, remains largely unknown. The aim of the present study was to build a model that explained the synchronization of activity in a multilevel society of feral horses. Multi-agent-based models were used based on four hypotheses: A) horses do not synchronize, B) horses synchronize with any individual in any unit, C) horses synchronize only within units, and D) horses synchronize across and within units, but internal synchronization is stronger. The empirical data obtained from drone observations best supported hypothesis D. This result suggests that animals in a multilevel society coordinate with other conspecifics not only within a unit but also at an inter-unit level. In this case, inter-individual distances are much longer than those in most previous models which only considered local interaction within a few body lengths.

## Introduction

Behavioural synchronization is the phenomenon where multiple individuals perform the same behaviours at the same time by mirroring each other, either consciously or unconsciously [1]. The patterns of synchronous activity have been found in many animals and with many different behaviours, from Placozoa to humans [2]. The common property of this collective behaviour is that relatively simple interactions among the members of the group can explain a global pattern of behaviour [3]. For example, a pattern of fission-fusion in some ungulate species could be simply explained by the dynamic tension between the advantages of aggregation and the disagreement among individuals, mainly between females and males, due to the variation in resource demand [4, 5]. Synchronization of behaviour is essential for animals to maintain the functions of a group and thus enhance their fitness and survival [1]. Fundamentally, animals need to synchronize the timing and direction of their movements to keep an aggregation [3]. Furthermore, it has been reported that synchronization can increase efficiency in their vigilance and defensive behaviours (like mobbing) against predators [6], as well as facilitating social interactions and enhancing social bonds [7, 8].

Many studies on synchronization were conducted on cohesive, single-layered groups, in either natural or experimental setups [6, 9–11]. In several social animals, social networks have a considerable effect on the propagation of behaviour [2, 12–16]. Socially central individuals can have a greater influence than subordinate individuals on group behaviour [17, 18]. Also, it was widely observed that socially affiliated dyads more intensely synchronized their behaviours [9, 19]. However, most studies which examined the social network effect were conducted on small, cohesive groups (but see [14]) whilst studies with large groups of individuals were based on anonymous mechanisms because of the difficulty in identifying and following all members.

Multilevel societies composed of nested and hierarchical social structures are considered to be among the most complex forms of social organization for animals [20–22]. In a multilevel society, the fundamental component is called a ‘unit’, and these units gather to form larger groups. While a higher-level social organization usually shows a fission-fusion pattern where units move independently and decide whether to join a temporal multi-unit group in the long term [23], they show collective and synchronized behaviour among different units when they form the multi-unit group [24, 25]. The most famous example of multilevel society is the troop, a third- or fourth-level social organization, of hamadryas baboons sleeping together on a cliff [26]. It is highly likely that synchrony occurs not only among the same units but also in a higher level of social organization, but studies on their synchronization mechanisms and functions are quite limited [27].

Multilevel society is characterised by a different association pattern in each social level. Usually, members of a unit stay close together, while the extent of cohesion becomes smaller as the social level increases [20, 23, 25, 28, 29]. Some studies have found that different units keep an intermediate distance from each other, staying farther apart than the inter-individual distance within units [30] but closer than random distribution [29]. It is argued that this differentiation of social relationships has evolved to balance the advantages of being a large group and the disadvantages of resource competition with other units [31–33]. For example, a study on golden snub-nosed monkeys (*Rhinopithecus roxellana*) suggested that harem unit aggregation could reduce the risk of inbreeding and bachelor threat, but being a large group may cause intense competition for food; hence, their aggregation pattern changes according to the seasonal prevalence of resources [23]. It was assumed that this fission-fusion pattern, which balances the competition and cooperation between units, could also be applied to behavioural synchronization. Whether multi-level societies show behavioural synchronization remains unclear, but it is important to address this question in order to better understand the collective features of such societies.

New technologies enable more wide-ranging and accurate data collection in societies with hundreds of individuals [34–36]. For instance, our use of drones succeeded in obtaining positional and behavioural data of a multilevel society composed of more than a hundred feral horses in Portugal. The research identified a two-layered structure of units (combinations of individuals that stayed closer than 15.5m over 70% of the time) nested within a herd (i.e., the observed inter-unit distance was significantly smaller than that of permuted data sets), and the permutation analysis confirmed that the herd formation consisting of multiple units was not because of confounding effects, such as resource distribution [29]. The current study further applies these data to investigate whether a horse multilevel society shows synchrony in resting/moving timing and, if so, whether the extent of synchronization changes within and across units.

**Fig 1:**
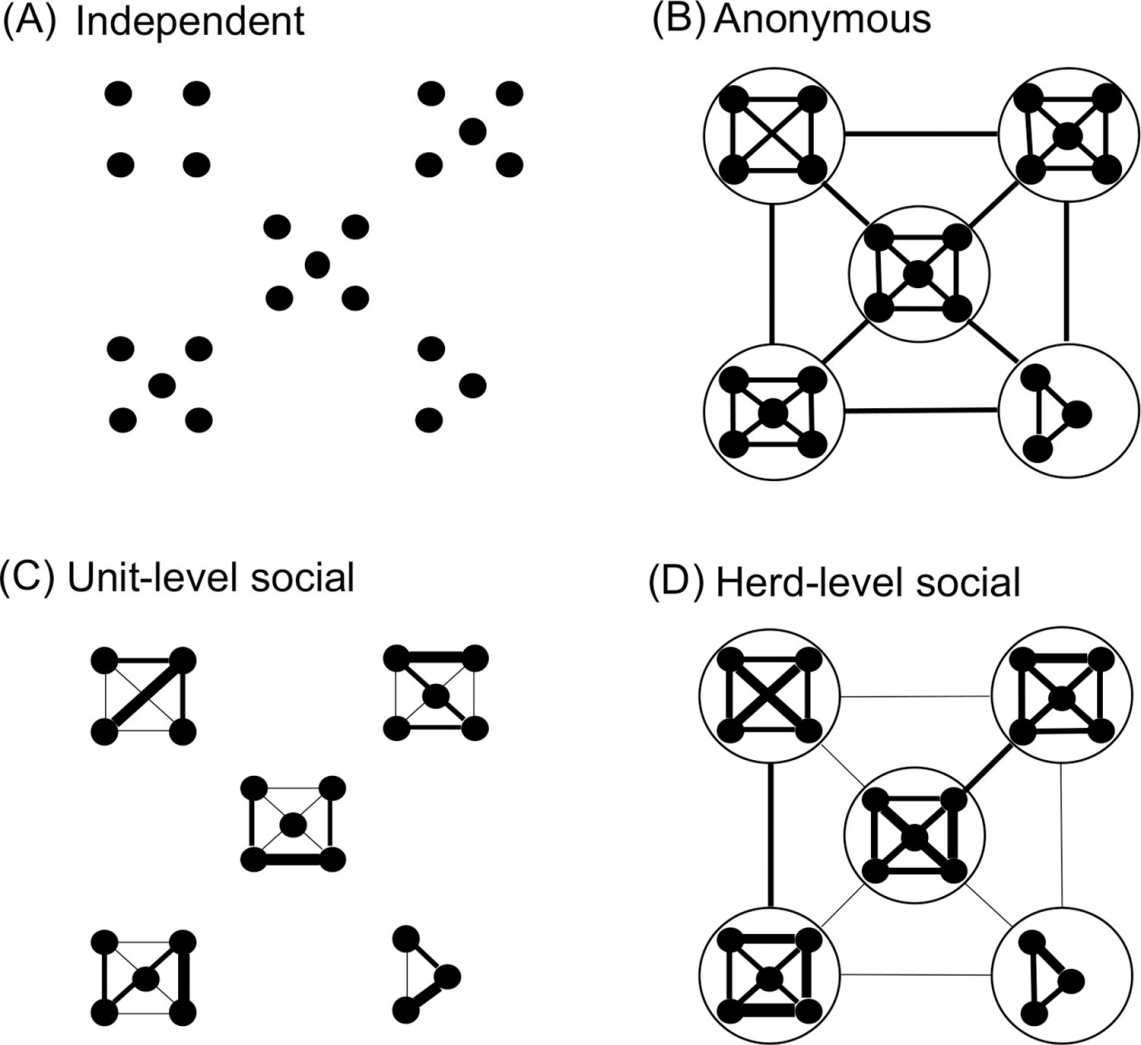
A graphic representation of synchronization models. The dots represent individual agents and the clusters of dots represent units. Agents/units connected with lines imply that their states were affected by each other. The width of the lines represents the strength of synchronization.

It is hypothesized that (1) horses synchronize their behaviour both at intra- and inter-unit levels and (2) the extent of synchronization in a dyad is correlated to its social relationships. In this study, different models were developed based on hypotheses ranging from no synchronization between individuals and units to full synchronization, with intermediate mechanisms based on social networks. In this way, this study develops a stochastic multi-agent-based model where the probability of an individual to change the stage (resting versus moving) depends on different hypotheses. (A) Independent: horses do not synchronize and are socially independent. This hypothesis is used as the null model. (B) Anonymous: horses synchronize with any individual in any unit. This hypothesis does not include the importance of stable social relationships in the trade-off between group-living advantages and competition. (C) Unit-level social: horses synchronize only within units, not considering the herd-level association and without the advantages of large societies. (D) Herd-level social: horses synchronize across and within units, but internal synchronization is stronger (Fig 1). Hypothesis D could achieve the best balance between the intra- and inter-unit level associations. Finally, these models are compared with empirical data to assess which models best explain synchronization in the present study’s population of feral horses.

## Methods

### (a) Data collection

Observations were conducted from 6th June to 10th July, 2018, in Serra D’Arga, Portugal, where approximately 150 feral horses were living without human care [37]. The field site had two large flat areas, Zone 1 and 2, which were visually separated by rocky hills (see Fig 3 of [29]). These areas were separated because horses moving between them during daytime was rarely observed. Drones (Mavic Pro: DJI, China) were used to accurately measure the distances between all individuals in the observation area of two zones covering approximately 1 km^2^ each. The flights were performed under clear sky conditions at an altitude of 30–50 m from the ground and successive aerial photographs of the horses present at the site were taken in 30-minute intervals from 9:00–18:00 (for more detailed explanation, see [29]). The average duration of each flight was 4 minutes 24 seconds ± 3 minutes 5 seconds. The field observations complied with the guidelines for animal studies in the wild issued by the Wildlife Research Center of Kyoto University, Japan (https://www.wrc.kyoto-u.ac.jp/guidelines/wild.html; in Japanese), and we signed a Memorandum of Understanding with the Viana Do Castelo municipality, which governs the study area and our field station. No further formal permission was required prior to conducting our research.

**Fig 2:**
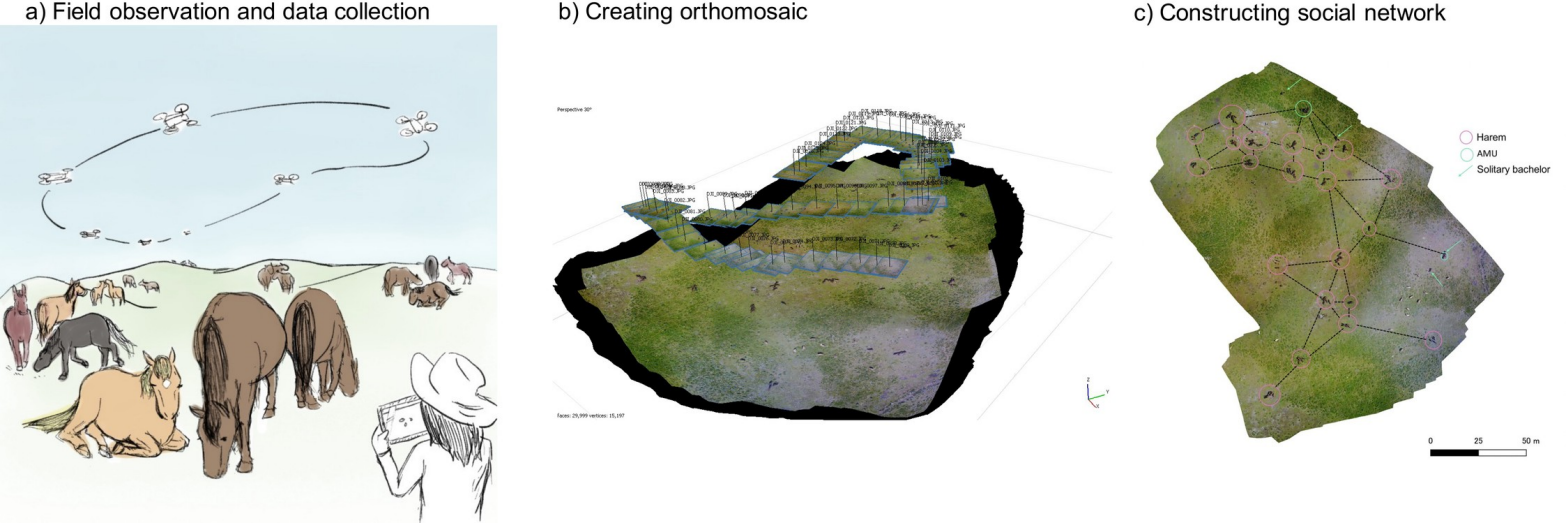
Overall research procedure. (a) Aerial photos of horses were taken using drones. (b) These successive photos were stitched together to create an orthomosaic. (c) Individuals in orthomosaics were identified, and the positional and behavioural data of horses were obtained. The social network was then constructed using inter-individual distance data. The photograph is also used in Maeda et al., (2021) published in Scientific Reports.

Orthomosaic imaging was conducted using AgiSoft PhotoScan Professional software. The software connected successive photos and created orthophotographs in the GeoTIFF format under the WGS 84 geographic coordinate system. All horses were first identified from the ground, following which an identification sheet was made for all individuals, recording their sex (whether they had testes); estimated age class; and physical characteristics such as colour, body shape, and white markings on the face and feet (Fig 2). The adults were individuals who experienced dispersal from their natal group, the young were those who were born in or before 2017 and still belonged to their natal group, and the infants were individuals born in 2018. All horses in the orthophotographs were identified accordingly. The heads of the horses were positioned and we recorded their resting status. The horses were considered to be resting if they did not move in the successive photos and showed a resting posture, that is, laying down or standing still with their neck parallel to the ground. Otherwise, they were considered to be moving. All locations were stored in shapefile formats. The coordinate system was converted to a rectangular plain WGS 84 / UTM Zone 29N and the distances were calculated between all pairs of individuals in the same zone. In total, 243 observations were conducted in 20 days and a total of 23,716 data points of individual positions were obtained (for detailed availability and number of observations each day, see S1 Fig). A total of 126 non-infant horses (119 adults: 82 females and 37 males; 7 young individuals: 6 females and 1 male) and 19 infants (11 females and 8 males) were successfully identified. They belonged to 23 units (21 harems and 2 AMUs; all-male unit), along with 5 solitary males. One adult female, named Oyama from Kanuma harem, disappeared sometime between the evening of 15th June and the morning of 16th June, probably preyed on by wolves. This female and two solitary males which were never located within 11m of other individuals were eliminated from the subsequent analysis. Infants were also eliminated because their position was highly dependent on their mothers.

### (b) Herd social network

**Fig 3:**
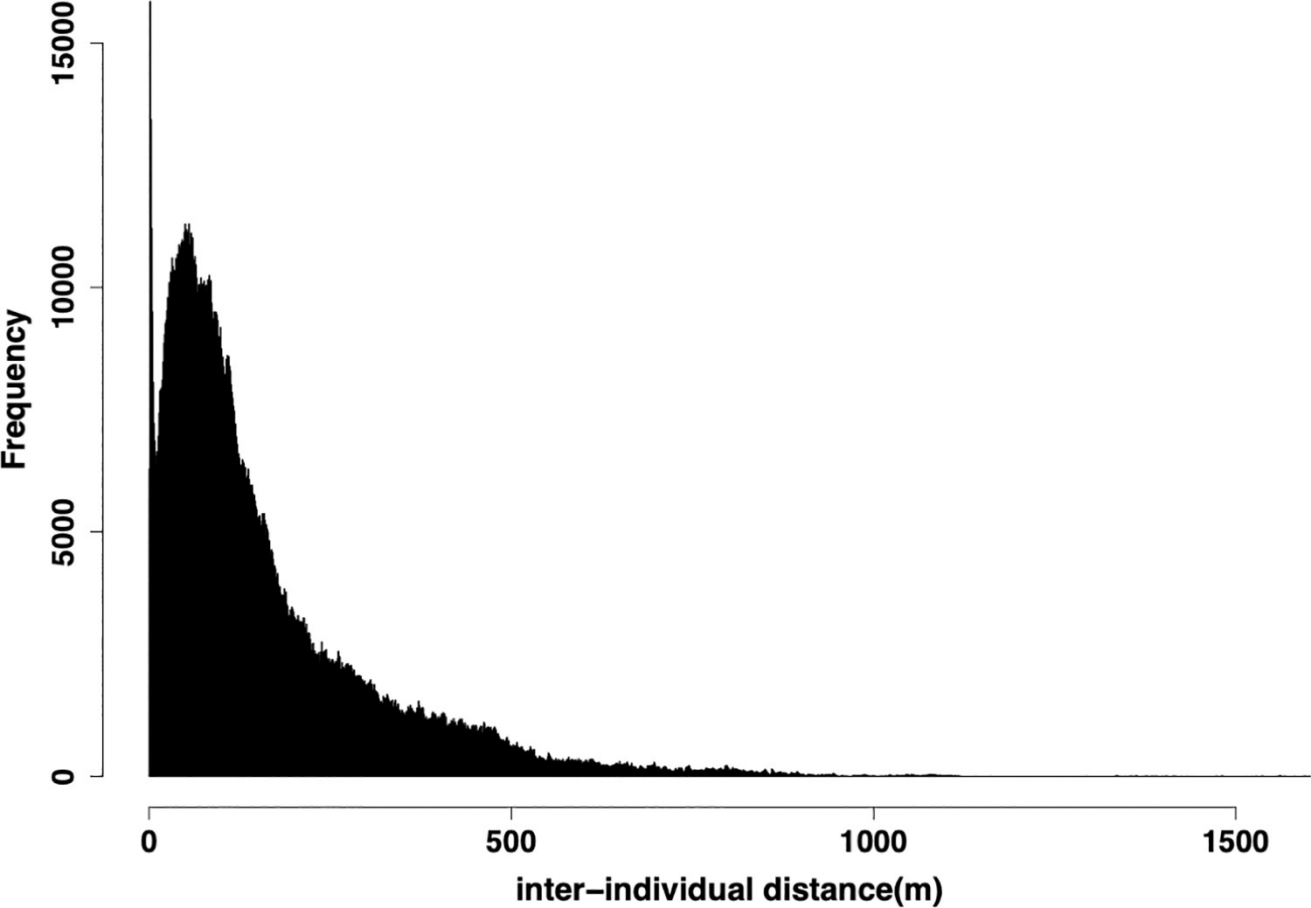
Histogram of inter-individual distances showing clear bimodality. The distance of the first and second peaks could be considered as the most frequent value of inter-individual distances within a unit and between units, respectively. The trough between two peaks represents the threshold that divides the intra- and inter-unit association. This figure is reprinted from Fig 2A in Maeda et al., (2021).

To create a social network, the threshold distance which defines the association was first decided. A histogram of inter-individual distance data was created under the R environment. The bin width was decided based on the method used in [38] and using R package ‘KernSmooth’ [39]. As shown in Fig 3, the histogram had two peaks–at the 2nd bin (0.9–1.8m) and at the 55th bin (49.7–50.6m) with a bin width of 0.92m. The minimum frequency, or nadir, between these two peaks was observed at the 12th bin (10.1–11.0m), and this was selected as the threshold distance that divides the intra- and inter-unit association (cf. [29]).

To obtain the social relationships for each dyad a_ik_, networks were generated for each sampling period (i.e., each flight of drones). Pairs of horses whose inter-individual distance was smaller than 11m were assigned an edge weight of 1, based on the threshold distance defined above. When two individuals were connected with each other indirectly via another individual, they were also considered to be connected (edge weight = 1). All other pairs were assigned an edge weight of 0. In the total number of drone flights, 658 temporarily isolated individuals who had no association with any other individuals were detected. If the distance from the nearest individual was smaller than *p*_2_ (the second peak of the histogram), it was presumed that they had an association with the nearest neighbour; otherwise, they were eliminated from the analysis. A total of 643 out of 658 isolated points were within 50.6m (the second peak of histogram) from the nearest individual. A social network was created from these co-membership data using the simple ratio index [40]. This calculates the probability that two individuals are observed together given that one has been seen, which is widely used in animal social network analysis. The density of the network was 0.047 ± 0.177 (average ± SD). The edge weight was normalized so that the sum of a_ik_ (k = 1,2,…, N; k≠i) became N (in other words, average network weight became 1.0).

### (c) Synchronization data scoring and calculation of modelling parameters

#### Population synchronization rate

At each time step (in this case, a scan every 30 minutes), the number N of horses and their identities in each state (S_r_ for resting and S_m_ for moving) were recorded. As explained previously, resting is standing still or laying down, and moving is any other behaviour, mainly grazing. Only observations when more than 90% (21 out of 23) of the units were available in the field were used. 21–23 units were observed in 8 out of 19 days during 14th-28th June and 5th July. The data from 5th July was not included, although 21 units were available, since horses foraged on the edge of the field site (rocky area with many obstacles), which may limit their vision. One AMU was not observed on the 15^th^ and 16^th^, and one harem and one AMU were not observed on the 28^th^ (S1 Fig). A synchronization rate of a dyad was defined as a proportion of the observation when two individuals were in the same activity state, that is, a score of 1 was given when two individuals were in the same state (e.g., resting or moving) in an observation and 0 when not, following which the average was calculated. During the daytime, the memberships of the multi-unit group rarely changed (one harem joined a group after observation started on 15^th^, 16^th^, and 18^th^ June).

#### Individual synchronization/state phase latency

**Fig 4:**
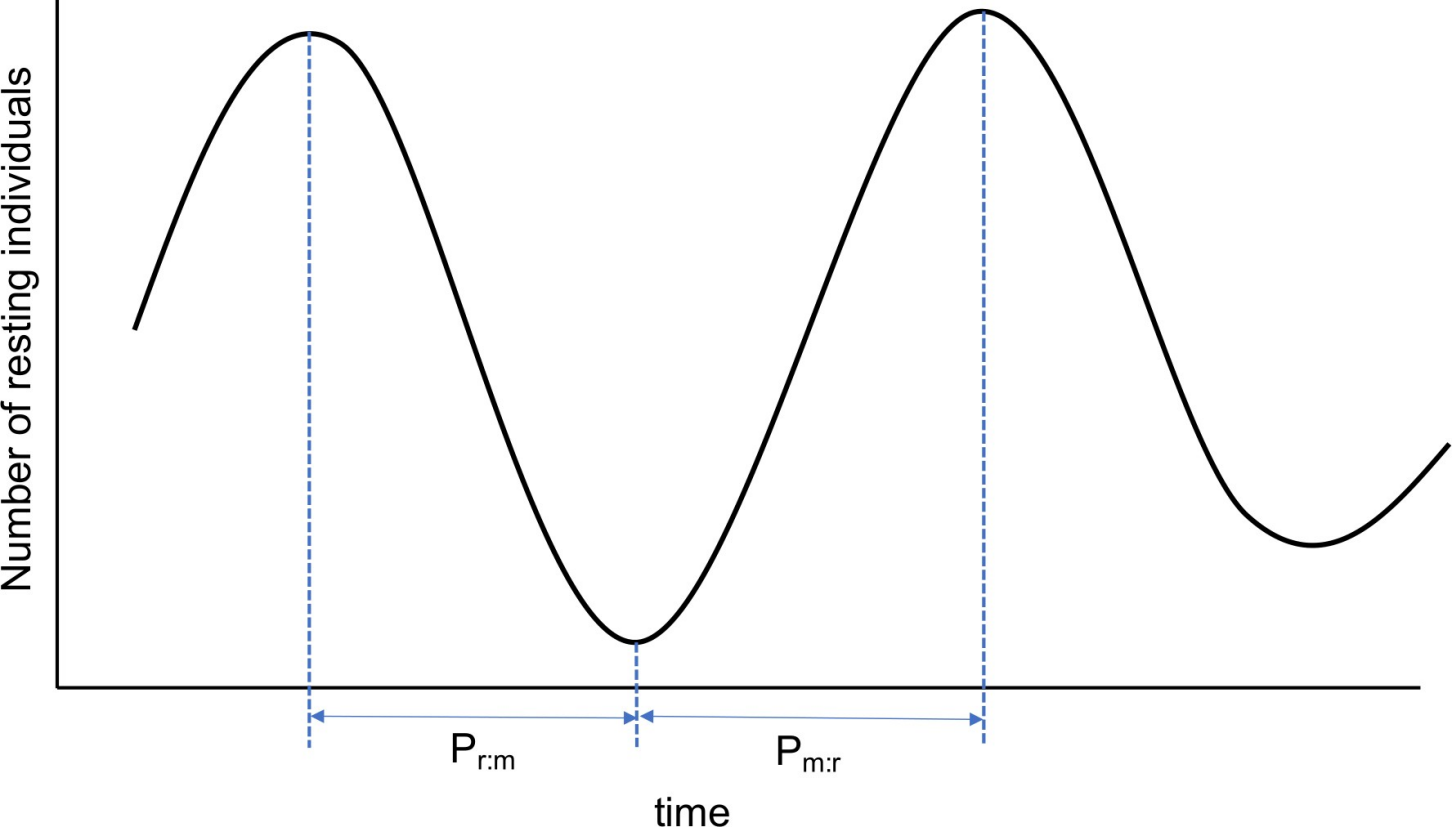
The explanation of P_m:r_ and P_r:m_.

A synchronization phase P_r:m_ was defined as a ‘resting → moving’ event when there was a continuous decrease of resting individuals from the minimal to the maximal and a phase P_m:r_ ‘moving → resting’ event as the opposite (Fig 4). The increase/decrease was excluded from the first or last observation of the day. In total, 21 moving → resting events and 18 resting → moving events were found. The state phase latency ΔT_01s_ was calculated as the time elapsed between the end of one state phase and the beginning of the next one. In previous works, this phase latency corresponds to the departure latency on an individual to change state [13, 17, 41, 42]. The value ΔT_01r_ corresponds to the resting phase latency and ΔT_01m_ to the moving phase latency (Table 1 and S1 Appendix). For explanations of modelling self-organisation and collectives, see also [13].

**Table 1:** The explanation and values of parameters.

parameter	value (resting)	value (moving)	Definition
a_ki_	social network		social network weight between individuals *k* and *i*.
C	0.426	0.796	mimetic coefficient
Ψ_1_	0.030	0.040	A probability of starting resting/moving. Equal to Nλ_i_
Ψ_j_	-	-	Probability per unit time that one of the n agents became the jth joiner (corresponding to the hypothesis where the identities of individuals are not taken into an account)
ψ_i,s_	-	-	Probability per unit time of an individual *i* changing the state s (the refractory time period and the identities of individuals are taken into an account.)
λ_i.s_	0.00016	0.00033	The average probability per unit time of an individual i changing the state s.
ΔT_01,s_	50 (min)	25 (min)	Refractory time period. time elapsed from the end of the previous event.
ΔT_j, j-1,s_	2.3 (min)	1.3 (min)	time elapsed between the state change of the joiner j-1 and the state change of the joiner j. The inverse of C.
Δt_i_	-	-	time elapsed from the previous state change of individual *i*
N	123		number of individuals in a herd
n_s_	-	-	Number of the individuals in the state s (resting/moving).

See also supplementary Table for the detailed explanation of how to obtain the parameter value. ‘-’ means that the value can change dynamically.

The value of the parameter was written when it was a constant.

#### Individual refractory period

Many synchronization processes in animal groups imply a refractory period, which is the short time period after an individual has changed their state and then appears insensitive to their neighbours [2, 43]. Theoretical studies showed that this period is necessary for animals to not be stuck in a state [2, 43], and preliminary works on our model also showed that, to avoid observing agents being stuck in a state, the refractory period is necessary. According to the observed data, the mean refractory period was 50 minutes for moving and 25 minutes for resting (S2 Fig). These values, as well as lower and higher values of the refractory period, were used to check the fitness of simulations to the empirical data (see supplementary material and section (d)). The changing state latency ΔT_j-1,j,s_ of each horse j changing state s corresponding to the time elapsed between the state change of the individual j– 1 was then scored, i.e., the previous individual changing state s1 to s2, and the state change of the horse j (changing also from s1 to s2). The expected value of ΔT_j-1,j,m_ and ΔT_j-1,j,_ were 2.3 and 1.3 minutes, respectively (Table 1 and Fig S2 in S2 Appendix).

The aims of this study were to understand the synchronization process of horses between two states—moving and resting—throughout the day. We considered that, in a multilevel society, individuals synchronize across and within units but their internal synchronization is stronger. In other words, the synchronization should be similar to their spatial association pattern, where intra-unit cohesion is quite strong but the inter-unit cohesion is moderate.

According to the preliminary analysis, the horses’ resting/moving was independent of the time of day (see S2 Appendix for detailed explanation); thus, the effect of time was not considered in the following models.

#### Model design

The overall design of the models is shown in Fig 1. The model is stochastic and individualistic [13, 43], signifying that the probability of each individual to change state, not the collective probability or state, is considered. This concept was followed as we introduced the selective mimetism (mimetism based on social relationships) as a hypothesis, and this can be done only by calculating probabilities per individuals [13, 17]. This bottom-up approach is also better than the top-down one for understanding individual decision processes. The probability of individuals to change states, mimetic-coefficient and refractory time period of resting/moving, and social relationships from the data set were obtained (Table 1, details about calculations are given below). The probability Ψ_1_ (*N*λ), mimetic coefficient C, and refractory time period ΔT_01_ (= 1/Ψ_1_) of moving were calculated as 0.04, 0.796, and 25 minutes and those of resting were 0.02, 0.426, and 50 minutes, respectively (Table 1 and S1 Fig). A simulation (one day) extending 9 hours (540 minutes) with 18 observations was conducted, and the simulations were repeated 100 times for each hypothesis. The model was also tested with different parameter sets to investigate its robustness. In most of the parameter sets, similar results were derived as detailed in the results section (S3 Appendix).

#### Individual probability of changing state

As the distribution of the state latencies corresponded to an exponential distribution (S2 Fig), the probability of an individual changing its state was the log gradient of this exponential distribution, that is, the inverse of the mean state latency [17]:

$$\Psi_{1,s}=\sum_{i=1}^{N}\lambda_{i,s}$$

It was assumed that all individuals may have the same mean latency, while their probability of changing their state might differ. The mean latencies to start an event are equal irrespective of the individual:

$${\Delta T}_{01,s}=\frac{1}{\Psi_{01,s}}$$

As explained above, *ΔT*_01_ was also defined as a refractory time period in the simulations.

#### Mimetic coefficient

**Fig 5:**
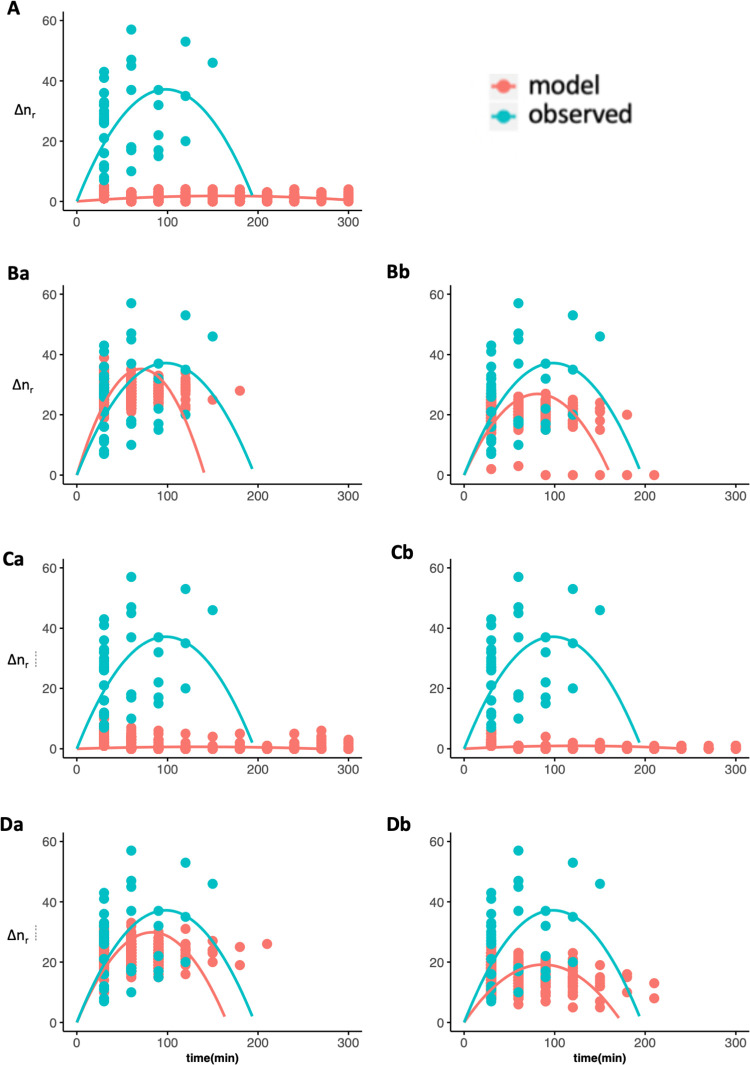
The change of the number of resting individuals in P_m:r_. The pink points are data obtained from simulation and blue are those from the observation. Data were fitted to a quadratic function that crosses (0,0), i.e., ax^2^+bx. R^2^ is the coefficient of determination of the regression for simulated data. Aa: independent, Ba: absolute anonymous, Bb: proportional anonymous, Ca: unit-level absolute social, Cb: unit-level proportional social, Da: herd-level absolute social, and Db: herd-level proportional social models.

**Fig 6:**
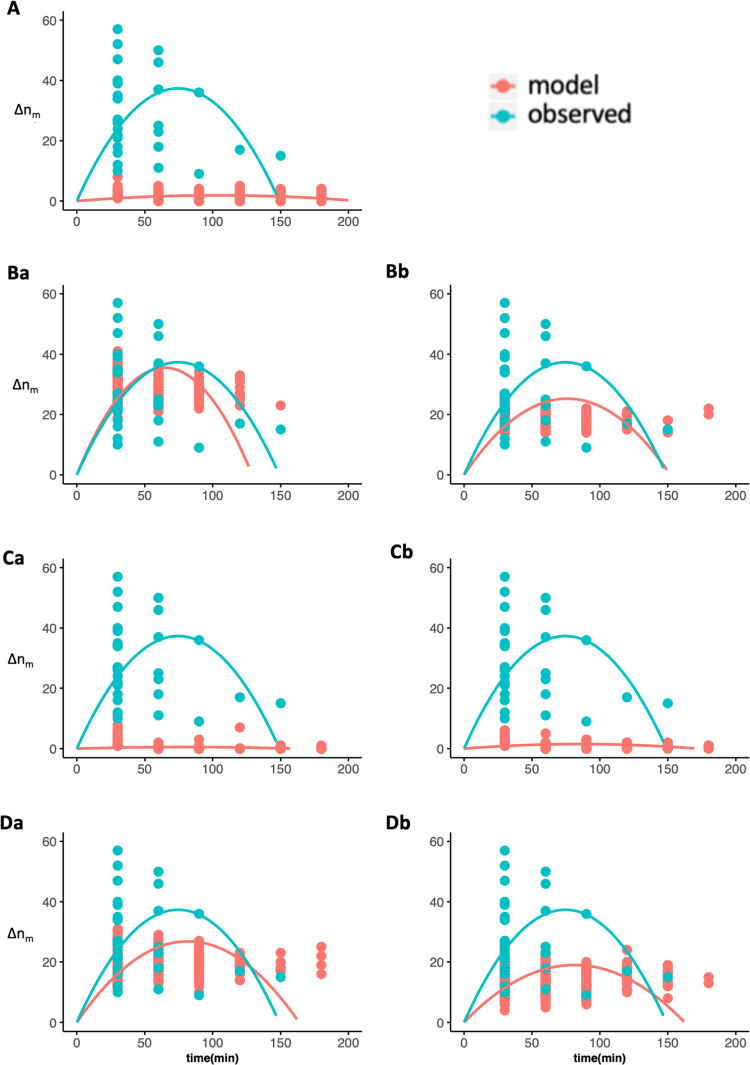
The change in the number of resting individuals in P_r:m_. Same as Fig 5.

In a mimetic process where the probability of changing state is proportional to the number of individuals already in this state, the probability per unit time that individual i changes state is as follows:

$$\psi_{i,s}=\lambda_{s}+Cn_{s}$$

where C is the mimetic coefficient per individual and j_s_ is the number of individuals in state s, either R for resting or M for moving. As *ψ*_*i*_ was the same for all the individuals in the herd, the mimetic coefficient C could be obtained from the inverse of the average T_j,j-1_, 1/E[ΔT_j,j-1_] (j = 2,3,…). The parameters C and *ΔT*_01_ were calculated using survival analysis (S2 and S3 Figs, respectively) and quadratic functions (see results and Figs 5 and 6).

#### Models based on the different hypotheses

Different sub-models (Fig 1) were tested based on each hypothesis, presented here for i to iii. Overall, seven models were tested: (A) independent, (Ba) absolute anonymous, (Bb) proportional anonymous, (Ca) unit-level absolute social, (Cb) unit-level proportional social, (Da) herd-level absolute social, and (Db) herd-level proportional social model.

*(i) Independent hypothesis (model A)*. The first hypothesis assumed that horses were independent: the probability of an individual changing their state is not influenced by the state of any other members. Under this hypothesis, the probability that one of the agents (e.g., individual i) changes state per unit time was *λ*_*i*,*s*_. Considering the refractory period, the probability ψ_*i*_ is equal to λ = *Ψ*_01_/*N* when Δ*t*_*i*_ *<ΔT*_*01*,*s*_ and is equal to 1 when Δ*t*_*i*_ *= ΔT*_*01*,*s*_. This model corresponds to a null model.

*(ii) Anonymous hypotheses (model Ba and Bb)*. The second hypothesis specified that horses synchronize with all the herd members anonymously. In the absolute anonymous model (model Ba), individuals will change state s according to the absolute (i.e., not proportional) number of herd members in that state (number R for state r and number M for state m). To test this hypothesis, a mimetic coefficient C was added in the independent model, which indicated the strength of the collective process.

Considering the refractory time period, the probability that one of n agents became the joiner j+1 in state s under the model Ba was obtained from the following equation:

$$\psi_{i,s}=min\{\frac{1}{{\Delta T}_{01,s}-\Delta t_{i}},\;\lambda_{s}+Cn_{s}\}$$

when Δ*t*_*i*_ *<ΔT*_*01*,*s*_. It is equal to 1 when Δ*t*_*i*_ *= ΔT*_*01*,*s*_ (this is the same for all the models; hence, we only refer to the probability when Δ*t*_*i*_ *<ΔT*_*01*,*s*_). The equation shows that when Δ*t*_*i*_ is small, that is, soon after an individual changed its state (beginning of a refractory period), it is less likely to be influenced by other individuals’ states.

Another model was created based on the proportional number of individuals in state s, where the probability of changing state s1 depended on the number of individuals in state s1 divided by the number of individuals in state s2 (model Bb). The probability of individuals in s2 to go in state s1 is as follows:

$$\psi_{i,s1}=min\{\frac{1}{{\Delta T}_{01,s1}-\Delta t_{i}},\;\lambda_{s1}+C\frac{n_{s1}}{n_{s2}}\}$$

As n_s1_ = N—n_s2_, the response of individuals become reciprocal, not linear like the anonymous model.

*(iii) Social hypothesis (model Ca*, *Cb*, *Da*, *and Db)*. In these hypotheses, the influence of the social relationships between units or herd members on the decision to join was tested. The unit-level social hypothesis (model Ca and Cb) assumed the synchrony happened only among unit members, while the herd-level social hypothesis (models Da and Db) considered both intra- and inter-unit sociality. Within these two social hypotheses, two models were tested: one taking the absolute numbers of individuals in each state (model Ca and Da), and the other taking the proportion as described for the anonymous mimetic models (Cb and Db).

Models Ca and Da considered the individual identities and the social relationships of each dyad. Each observed social relationship of the study herd was implemented in the model, allowing the consideration of differences in social relationships between dyads. The probability per unit time that one of the n_s2_ individuals would change the state to n_s1_ differed inversely between the resting agents with respect to their social relationships with agents already in s1. The probability of an individual i to change state under the social hypothesis was as follows:

$$\psi_{i,s}=min\{\frac{1}{{\Delta T}_{01,s}-\Delta t_{i}},\lambda_{s}+C\sum_{k\in s,k\neq i}a_{ik}\}$$

where *k*∈*s* means that individual k is in state s. We simulated two types of the social index a_ik_: ‘unit-level’ (only intra-unit) in model Ca, and ‘herd-level’ (both intra- and inter-unit) association network in the model Da to investigate whether individuals made decisions based only on the members of the same unit or on all herd individuals.

In models Cb and Db, the proportion of the joiner to the non-joiner mattered. The probability of an individual i becoming a joiner j+1 under the social hypothesis was as follows:

$$\psi_{i,s1}=min\{\frac{1}{{\Delta T}_{01,s1}-\Delta t_{i}},\;\lambda_{s1}+C\frac{\sum_{k\in s1,k\neq i}a_{ik}}{\sum_{k\in s2,k\neq i}a_{ik}}\}$$

#### Model setup

At the group level, the collective state S(t) can be described at time t by the number n_m_ of individuals moving at that time (for a given group size N, the number n_r_ of resting individuals is always N—n_m_).

The number of individuals, individual identities, and social relationships of the observed herd were included in the model. Thus, the number of agents N was fixed to 123. The model is time-dependent with each time-step representing one minute. At the start of the simulation, 30% of the agents were resting (n_r_ = 37). This 30% was derived from the average percentage of resting horses through observation. This value was consistent with the other studies of feral horses [44]. The probability of changing state *λ*_*i*_ of each agent was implemented. The present study did not implement any ecological barriers in the model, as the horses usually foraged in a flat area with almost no obstacles (i.e., trees or rocks).

### (e) Statistical analyses

To evaluate the model fit, this study compared the number of horses changing states and the synchronization rate of simulated data to those of observed data.

For both P_m:r_ and P_r:m_, the number of individuals which changed state after the synchronization phase started in each 30-minute window (e.g. 0–30, 30–60, 60–90 min…) was plotted. We refer to this number as Δn_s_ (Δn_r_ is for P_m:r_ and Δn_s_ for P_r:m_). The observed data were fitted to a quadratic function that crosses (0,0), that is, ax^2^+bx, using linear regression in the R environment. The models were evaluated by comparing the simulated data to observed data using the Kolmogorov-Smirnov (K-S) test.

The correlation between the synchronization rate per dyad of simulated data and that of observed data was calculated, and its significance was tested using the Mantel and K-S tests. The similarity of the intra-unit synchronization rate distribution was evaluated to that of the observed data using the K-S test. The Mantel test was used to evaluate the similarity of the synchronization rate matrix as a whole, especially the ratio of intra- and inter-unit synchronization rate. Indeed, the synchronization rate across units were mostly the same among models and never became better than independent; therefore, it was eliminated from the evaluation. A Mantel test was performed using the R package ‘vegan’ [45] and K-S tests were performed using the function ‘ks.test’ in the R environment.

Horses live in a multilevel society and are therefore expected to show social cohesion and behavioural synchronization. It was thus expected that the mimetic model, either anonymous or social, would do better than the independent model (model Aa). Thus, the model Aa was defined as a null model and other models were compared to the same. A score for each model was calculated, defined as the proportion of the model showing better results than the independent one, that is, when the model had lower D in K-S tests and higher r in Mantel tests than those of the independent model. As we had four tests, the score takes a value, 0, 0.25, 0.5, 0.75, or 1.0, where 1.0 is regarded the best.

## Results

### (a) Empirical data

The average number of individuals changing states are shown in Figs 4 (P_m:r_) and 5 (P_r:m_) (in blue, repeated in all graphs for comparison). Both showed a positive correlation with the quadratic function (adjusted R^2^ = 0.79 in P_m:r_, R^2^ = 0.81 in P_r:m_, see S1 Table for the detailed results), indicating a mimetic or synchronization process with an increase of the number of horses in a state followed by a decrease [13, 17].

The average ± SD synchronization rate of each pair was 0.93 ± 0.03 within unit and 0.63 ± 0.06 across units in the observed data, which showed a strong synchronization based on the social network of horses. The correlation of the social network and synchronization rate of observed data was 0.69 (Mantel test, permutation: 9999, p<0.001), indicating a synchronization process based on social relationships, but a part of the process (at least 31%) was not based on these relationships.

The average ± SD weight within and across units was 19.4 ± 9.9 and 0.26 ± 0.85, respectively. This suggests that the same unit members had around 75.9 times stronger effects on the behaviour than horses from different units in the herd-level hypothesis, and as units are mixed (different ages, sex, and personality), other hypotheses (sex, age, and personality tested separately from the network) are not relevant compared to the social network which embeds these sociodemographic variables.

### (b) Simulations

Concerning the states’ synchronization, four models showed parabolic shape correlated to observed data (Table 2) in the moving to resting phase (absolute anonymous: Fig 5Ba, proportional anonymous: 5Bb, herd level absolute social: 5Da, and herd-level proportional social: 5Db) and resting to moving phase (absolute anonymous: Fig 6Ba, proportional anonymous: 6Bb, herd level absolute social: 6Da, and herd-level proportional social: 6Db). Agents merely changed their states in the other three models (independent: Figs 5A and 6A, unit-level absolute social: 5Ca and 6Ca, and unit-level proportional social: 5Cb and 6Cb).

**Table 2:** The result of the evaluation of Δn and the synchronization rate obtained from the simulations.

		K-S test (Δn_m_, P_r:m_)			K-S test (Δn_r_, P_m:r_)			K-S test (sync rate of intra-unit)			Mantel test (sync rate)			
model		D	p	eval	r	p	eval	D	p	eval	r	P	eval	score
Independent (null model)	A	0.980	0.010		0.980	0.010		1.000	<0.001		0.367	0.010		
absolute anonymous	Ba	0.215	0.989	+	0.306	0.989	+	1.000	<0.001	-	-0.050	0.989	-	**0.50**
proportional anonymous	Bb	0.564	0.947	+	0.561	0.947	+	1.000	<0.001	-	-0.057	0.947	-	**0.50**
unit-level absolute social	Ca	0.993	<0.001	-	0.995	<0.001	-	0.973	<0.001	+	0.572	<0.001	+	**0.50**
unit-level proportional social	Cb	0.980	<0.001	-	0.980	<0.001	-	0.993	<0.001	+	0.627	<0.001	+	**0.50**
herd-level absolute social	Da	0.537	<0.001	+	0.577	<0.001	+	0.915	<0.001	+	0.601	<0.001	+	**1**
herd-level proportional social	Db	0.698	<0.001	+	0.714	<0.001	+	0.714	<0.001	+	0.634	<0.001	+	**1**

“Eval” (evaluation) is “+” when the result is better than the independent model and “-” when not. The model with smaller D and larger r is considered as better. Score is the percentage of the tests which showed better results than the independent (null) model.

**Fig 7:**
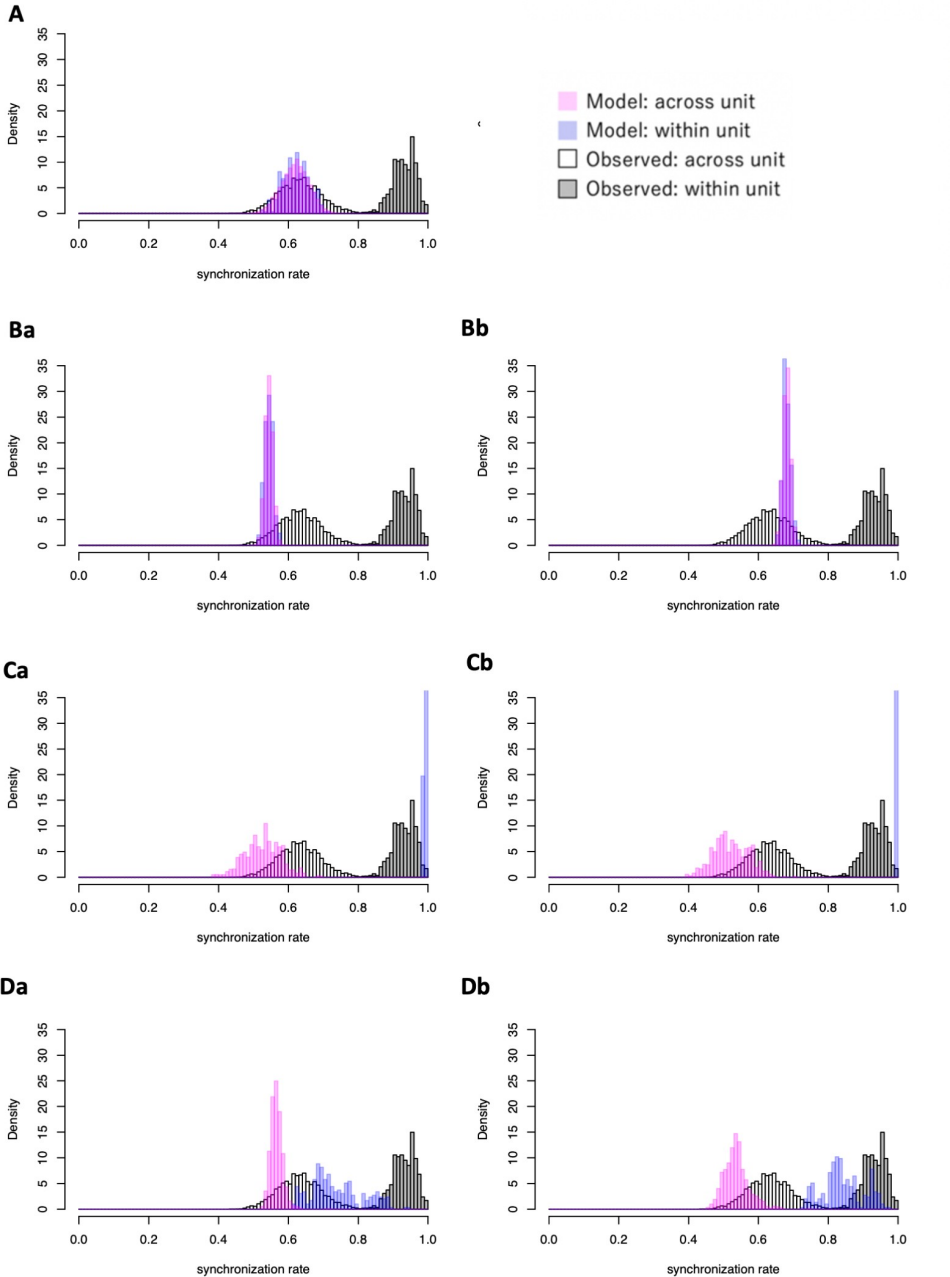
Histograms of the synchronization rate. White and grey bars represent the observed value of synchronization rate across units and within units, respectively. Pink and blue bars represent those of the simulated data across units and within units, respectively. The name of the models is same as in Fig 5.

Fig 7 shows the comparison between model-generated synchronization scores and synchronization scores from the empirical data. The model simulations that did not consider social relationships (i.e., independent, absolute anonymous, and proportional anonymous models) showed a lot of overlap in the histograms of intra-unit and inter-unit synchronization scores, unlike the observed data, which show clear separation between intra and inter-unit synchronization scores (Fig 7).

Overall, the herd-level social (model Da) and proportional social (model Db) always had better scores than the independent (null) model, while the others did not. The K-S tests for Δ*n*_*m*_ and Δ*n*_*r*_ were better in the herd-level social model, and the K-S test and the Mantel test were better in the herd-level proportional social model (Table 2).

## Discussion

The present study compared seven models to find one that best explained the dynamics of behavioural states, specifically the synchronization of resting versus movement, in horses’ multilevel society. Among the models tested, only the herd-level absolute social model (model Da) and the herd-level proportional social model (model Db) matched the empirical data better than the null model (model A). Considering the simplicity of the model, which does not contain any environmental effects and temporal changes of agents’ positions, and the fact that the model is based on temporally sparse data with 30-minute intervals, we argue that these two models were quite fitted to the empirical data. These models indicate that synchronization in a multilevel society of horses can be largely explained by their internal rhythm plus the social network. Model Da (herd-level absolute) was better at explaining the number of horses changing states, while model Db (herd-level proportional) was more successful at explaining the synchronization rate distribution; thus, the mechanism most likely lies somewhere between them (for instance, these two mechanisms switch at a certain threshold). It is also possible that the fitness of the two models could not be evaluated with sufficient accuracy because of the sparsely observed data. Although a multilevel society is considered among the most complex social structures in animals [22], this study suggested that the collective behavioural pattern could be represented by simple mathematical models.

The observation data had higher intra- and inter-unit synchronization rate, and the number of individuals that change state after the synchronization phase started (Δn_r_ and Δn_m_) had a higher peak than those of the herd-level hypothesis (models Da and Db) in most of the parameter sets. The Δn_s_ represents the speed of the behaviour spread, and synchronization rate corresponds to the stability of the state (e.g., whether horses keep resting when many individuals are resting), suggesting that both are stronger in the observed data than those in simulation. According to the models with different parameter sets, the fitness to Δn_s_ value and to the synchronization rate was negatively correlated with each other, suggesting the trade-off between them (S3 Appendix). Indeed, the higher the speed of synchronization, the lower the stability. To further improve the fitness of the model, parameter sets and/or equations which establish compatibility between speed and stability may need to be considered. For example, in the current model, a shorter refractory time period could enhance the speed but lower the stability, because agents will definitely wake up after the refractory time passes. We may need to either change the equation of the refractory time period or enhance the speed without changing the refractory time period.

Most previous studies of non-multilevel societies suggested local interaction within a few body lengths or the several nearest neighbours [3]. However, the result from this study showed that inter-individual interaction also occurred among spatially separated individuals. According to [29], the average nearest unit distance was 39.3m (around 26.2 times a horse’s body length) and the nearest individual within the same unit was 3.2m. It is still unclear whether horses have a global view or if they just respond to the several nearest units; however, this is a notably large distance compared to other studies. Horses usually do not create any significant cue (e.g., vocalization) when they start moving/resting; thus, it is likely that horses have an ability to recognize the behaviour of horses of both the same and other units simultaneously. In a multilevel society, it is important to keep the inter-unit distance moderate. This avoids competition between units while keeping the cohesion of the higher-level group to obtain the benefits of being in a large group, such as protection from bachelors or predators [24], and may have led to the evolution of such cognitive abilities.

Besides the temporal positions of units, another factor which may be important is the individual and unit attributes. The integration of the network in the model already considered individual differences in network connectedness and centrality caused by such variations in attributes. In the intra-unit level, some individual characteristics could affect the leaderships of collective departure in a multilevel society (lactation: [46]; personality: [19]; intra-unit dominance rank: [14, 47]), but it is unclear if those factors affect the behavioural propagation in the herd level (but see [46]). We presume most of these individual-level attributes would become less effective in inter-unit level synchronization because each unit has individuals with a different status, and the synchronization inside units are far stronger than those across units. In herd-level synchronization, it may be assumed that all individuals in the same units always perform the same behaviour (all resting or all moving); thus, individual differences should be largely diluted. However, it is still possible that a unit-level social status exists and affects the synchronization pattern. In this horse population, our previous study found that large harems tend to occupy the centre and had higher strength centrality (the sum of the edge that connects to a node), while small harems and AMUs stayed on the periphery, suggesting the existence of inter-unit level dominance rank [29]. It may therefore be possible that such dominant units are more influential. Our data were too sparse in time scale to observe how behaviour propagate across units and include horses’ positional dynamics in a model, which is needed to investigate horses’ recognizable distance and the effect of the attributes. Finer-scaled observation will be needed for further investigation on the underlying mechanism in herd-level synchronization.

Because of the simplicity of the model, the present methodology is highly applicable to other species. The spatial structure of multilevel societies is still poorly understood, but it may vary among species, habitat environments, and contexts. For example, a migrating herd of Prezewalski’s horses (*Equus ferus przewalskii*) was relatively aggregated [48], but a higher-level group of Peruvian red uakari (*Cacajao calvus*) was much more sparsely distributed, similar to the horses in this study (the nearest unit distance was 10–15m or more) [30]. It is also highly possible that other species forming multilevel societies show an ability to recognize the behaviour of other units which are located far away (especially in species that live in open fields, such as equines and cetaceans). Horses do not have specific timing for resting, and it is unlikely that all individuals sleep at the same time. Thus, to test whether agents only perceive units nearby, a formula representing collective movement needed to be added in the current model. However, some animals that form multilevel societies, such as primates, often sleep together at the same location at night [20]. In that case, the movement need not be considered, making it easier to test the range of their perception. It is important to discover whether the association index could also explain the behavioural decisions of other multilevel social animals with various special structures to generalize our knowledge of behavioural synchronization in multilevel societies.

Overall, this study provides new insights into the behavioural synchronization process and contributes to the understanding of collective behaviours in complex animal societies. The organization of multilevel societies has become a topic of great interest recently, but studies have so far tended to focus on social relationships, and many questions remain unresolved. It is hoped that this study on collective synchronization will contribute to an understanding of the evolution and functional significance of multi-level animal societies.

### Limitations of the study

Our model could not consider the temporal changes in the position of horses, including concurrent inter-individual and inter-group distances, although it is highly likely that the behaviour of units is more affected by closer units. While horses are in the moving state, their movement is likely to be synchronized with each other; hence, movement synchronization as well as behavioural state synchronization may need to be considered in a model. Inter-individual and intergroup distances can be developed in the model indirectly through giving variance using stochasticity to relationships implemented in the model. For calculating the parameter on stochasticity, more temporally fine-scaled data may be needed. Orthomosaic data have the advantage of obtaining accurate and identified positions of individuals in a wide-ranged group, but it obtains only temporally sparse data. Optimizing the data collection method, such as a combination of the video recording from drones and orthomosaics, is needed to further develop the model. In addition, the variations of parameter sets tested in this study were limited, making it difficult to hold a detailed discussion on the function of the parameters.

## Supporting information

S1 Appendix Reliability test of behavioural identification from drones. (PDF)

S2 Appendix The effect of time on the horses’ behavioural state. (PDF)

S3 Appendix Comparisons of models under various parameters. (PDF)

S1 Table The result of the regression analysis of Δns in observed data. Data was fitted to quadratic function that cross (0,0), i.e. ax^2^+bx.
(DOCX)

S1 Fig The number of observations per day and unit availability. The orange cells indicate the day we used in the analysis.
(PDF)

S2 Fig Survival analysis to obtain (a) ΔT_01,m_ and (b) ΔT_01,r_. The data was fitted to exponential curve. The absolute value of the exponent is considered as the inverse of ΔT_01,s_ (minutes).
(PDF)

S3 Fig Survival analysis to obtain mimetic coefficient C for (a) P_r:m_ and (b) P_m:r_. The data was fitted to exponential curve. The absolute value of the exponent is considered as C, which is the inverse of ΔT_j,j+1,s_ (minutes).
(PDF)
