# Supplementary material for: Behavioural synchronization in a multilevel society of feral horses
Source: PLoS One. 2021 Oct 26;16(10):e0258944. doi: 10.1371/journal.pone.0258944 (PMC8547633; doi:10.1371/journal.pone.0258944)
Supplement: S1 Appendix — (PDF) [file pone.0258944.s001.pdf]

S1 Appendix

Reliability test of behavioural identification from drones.

We examined the accuracy of our identifications of resting/moving states. On 17, 21, 22, and 24 June and 10 July 2019, we took orthomosaics of the herd and later speculated upon their behaviour. At the same time, we selected one unit randomly and did a focal sampling of the unit throughout a day. Their behaviour was recorded using a video camera from the ground level. When a horse continued the resting posture for more than 1 minute, we considered the horse to be resting. We used this data as a correct answer.

As a result, we took 44 orthomosaics and recorded 204 behaviours of 4 units (18 individuals). The correct answer rate was 99.0%. The identification of behaviour from drones were found to be sufficiently reliable.

	**eating**	**resting**	**total**
correct	172	30	**202**
incorrect	0	2	**2**
**correct answer rate**	1.00	0.94	**0.99**

Table S1_1. The result of reliability test in behavioural identification from drones.
