# Supplementary material for: Behavioural synchronization in a multilevel society of feral horses
Source: PLoS One. 2021 Oct 26;16(10):e0258944. doi: 10.1371/journal.pone.0258944 (PMC8547633; doi:10.1371/journal.pone.0258944)
Supplement: S2 Appendix — (PDF) [file pone.0258944.s002.pdf]

S2 Appendix

The effect of time on the horses' behavioural state

We first examined whether the resting/moving behaviour of horses had a temporal periodicity. We fitted the moving rate to the periodic function $y \sim x + \sin 2\pi x + \cos 2\pi x$, where x is a time, with a random effect on the intersect, which is the observation date, using linear mixed model. The model was compared to null models, $y \sim x$ and $y \sim (constant)$, using analysis of variance (ANOVA). The analysis was conducted under R package 'lme4' and 'lmerTest'.

The ratio of moving individuals according to time is shown in Figure S2_1. As a result, the fitted model is no better than the null models (Table S2_1). In addition, none of the model coefficients had a 5%-level significance. We concluded that the horses did not rest or move at a particular time of day, and thus we did not consider the effect of time in further analysis.

	AIC	BIC	logLik	deviance	Chisq	Df	p value
y~(constant)	-50.777	-42.648	28.388	-56.777			
y~x	-52.189	-41.351	30.094	-60.189	3.4119	1	0.06473
y~x+sin2πx+cos2πx	-49.049	-32.792	30.524	-61.049	0.8603	2	0.6504

Table S2_1. **The result of ANOVA test**. logLik = log likelihood, Chisq = Chisquare.

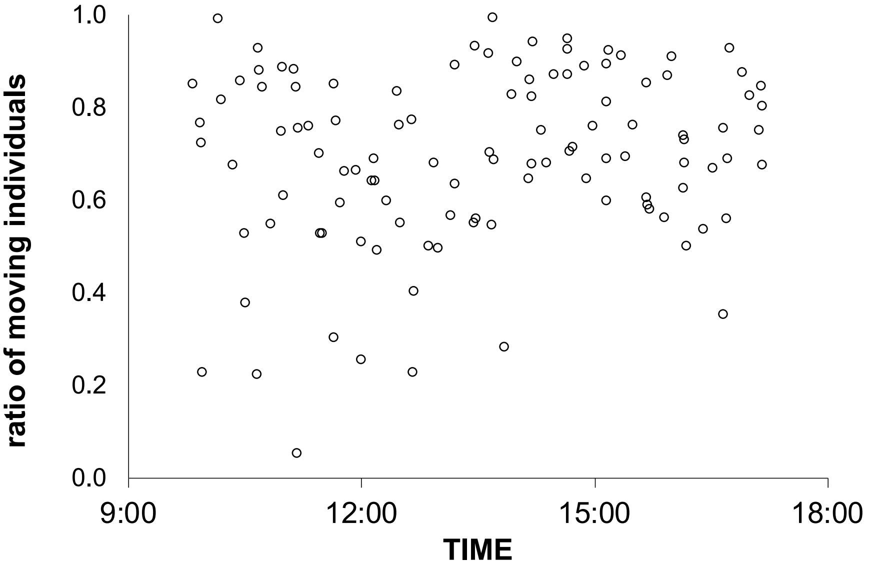

Fig S2_1. The ratio of moving individuals versus time.
