# Supplementary material for: Behavioural synchronization in a multilevel society of feral horses
Source: PLoS One. 2021 Oct 26;16(10):e0258944. doi: 10.1371/journal.pone.0258944 (PMC8547633; doi:10.1371/journal.pone.0258944)
Supplement: S3 Appendix — (PDF) [file pone.0258944.s003.pdf]

S3 Appendix

Comparisons of models under various parameters

We compared the models under various parameter sets to evaluate the robustness of the result. The parameter sets are shown in Table S3_1.

The new mimetic coefficients C = (0.955, 0.711) were created based on the same data but a different calculation. In the original model, we used the exact number of the horses changing states. However, not all the units were available in the observation site in some days, which may cause underestimation of the parameters. For the calculation of the new mimetic coefficients, we first calculated the proportion of the individuals' changed states and then multiplied by 123, the maximum number of individuals.

We also tested the robustness of the network structure by dividing the observation into two subsets. The original network was calculated using all the observations (19 days), but other parameters (mimetic coefficient, refractory time period, etc.) were created from the days when more than 21 units were available (8 days). We created a new network from the 8 days and the remaining 11 days using the same method as in the original one. We evaluated these models using the same method explained in the subsection (e) Statistical analyses in the Method section of the main manuscript.

The overall tendency did not change when we altered mimetic coefficients and refractory time periods (Table S3_2). When compared to the other models, the model Da (herd-level absolute social) and Db (herd-level proportional social) always showed the best score (1.0) except one, which means that they had better results than the null model (independent model with the original parameters) in all four tests. However, when we changed the proportion of the association index (increased inter-unit scores in the parameter set 10), the score of the model Da and Db became lower than 1.0. The result indicates that the model is robust to the changes of mimetic coefficients and refractory time periods, but susceptible to changes in the proportion between intra- and inter-unit association index. Moreover, the results were consistent when we separated the networks into two different subsets of observations (parameter sets 11 and 12). The results indicate the stability of the relationships among horses during the observation period. The result was also consistent with our previous study, which suggested that units were not just randomly associated with others, or that they were associated with certain units.

In the following analysis, we only refer to the model Da and model Db. We calculated the correlation among the scores of four tests with R package 'Hmisc'. As a result, two tests on synchronization rate

and those on Δn_s negatively correlate with each other.

	refractory time period ($\Delta T_{01,s}$)		mimetic coefficient (C)		association index(a_{ki})
	resting	moving	resting	moving	
original	50 (min)	25 (min)	0.426	0.796	
1	25	13			
2	100	50			
3	200	100			
4	25	50			
5	100	25			
6	100	37			
7			0.955	0.711	
8	25	13	0.955	0.711	
9	100	50	0.955	0.711	
10*					inter-unit affiliation ×10
11*					network created from 8 days when 21 or more units were available
12*					network created from 11 days when less than 21 units were available

Table S3_1. Different parameter sets. When the cell is empty, it means that the value is same as the original parameter sets. *Only herd-level linear association and herd-level proportional association model were tested.

	model						
parameter sets	A	Ba	Bb	Ca	Cb	Da	Db
1	0.25	0.5	0.5	0.5	0.75	1	1
2	0	0.5	0.5	0.5	0.75	1	1
3	0.25	0.5	0.5	0.75	0.75	1	1
4	0.5	0.5	0.5	0.5	0.5	1	1
5	0.25	0.5	0.5	0.5	0.75	0.75	1
6	0.25	0.5	0.5	0.5	0.75	1	1
7	0.5	0.5	0.5	0.5	0.75	1	1
8	0.25	0.5	0.5	0.5	0.75	1	1
9	0.25	0.5	0.5	0.75	0.75	1	1
10	-	-	-	-	-	0.5	0.75
11	-	-	-	-	-	1	1
12	-	-	-	-	-	1	1

Table S3_2 The evaluation of the models with different parameter sets. The numbering of the parameter sets correspond to that of Table S2.

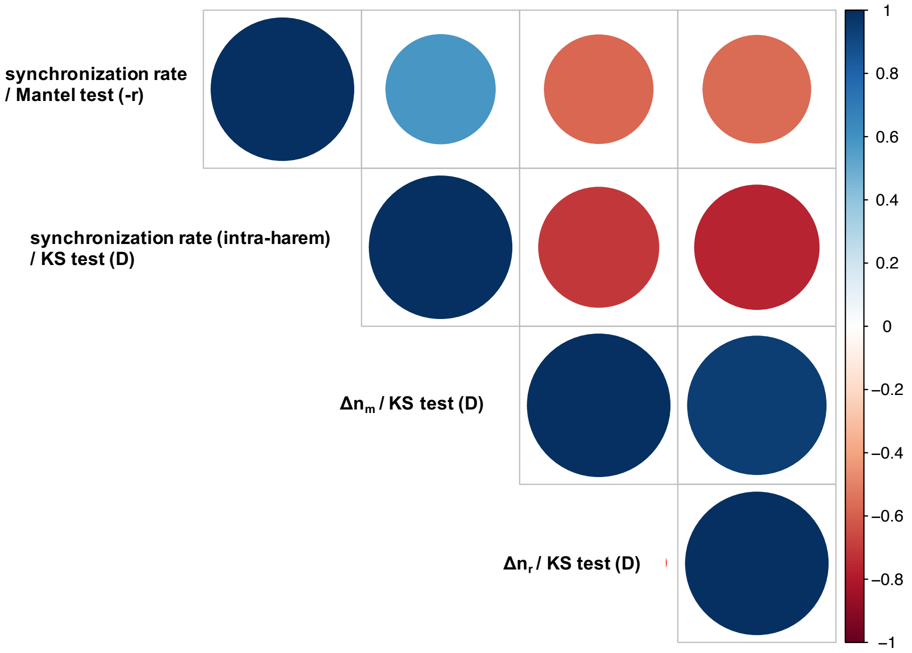

Fig S3_1. The result of the correlation test. The colour and the size of the circle corresponds to the correlation coefficients. We converted correlation coefficient of Mantel test to minus. All the correlation were significant at 1% level. The graph was created using R package 'corrplot'.
