# Supplementary material for: Behavioural synchronization in a multilevel society of feral horses
Source: PLoS One. 2021 Oct 26;16(10):e0258944. doi: 10.1371/journal.pone.0258944 (PMC8547633; doi:10.1371/journal.pone.0258944)
Supplement: S1 Table — Data was fitted to quadratic function that cross (0,0), i.e. ax2+bx. (DOCX) [file pone.0258944.s004.docx]

**S1 Table. The result of the regression analysis of Δn_s_ in observed data**. Data was fitted to quadratic function that cross (0,0), i.e. ax^2^+bx.

|  | Δn_m_ (P_r:m_) | | | |  | Δn_r_ (P_m:r_) | | | |
| --- | --- | --- | --- | --- | --- | --- | --- | --- | --- |
|  | Coefficient | SE | t value | p value |  | Coefficient | SE | t value | p value |
| a | -0.01506 | 0.004291 | -3.509 | 0.0016 |  | -0.003857 | 0.0009473 | -4.071 | 0.000236 |
| b | 1.39200 | 0.21584 | 6.454 | 6.46E-07 |  | 0.757462 | 0.095183 | 7.958 | 1.56E-09 |
