# Supplementary material for: Behavioural synchronization in a multilevel society of feral horses
Source: PLoS One. 2021 Oct 26;16(10):e0258944. doi: 10.1371/journal.pone.0258944 (PMC8547633; doi:10.1371/journal.pone.0258944)
Supplement: S1 Fig — The orange cells indicate the day we used in the analysis. (PDF) [file pone.0258944.s005.pdf]

Date	Number of observations	Number of units	Number of harem	Number of AMU	solitary male1	solitary male2	solitary male3
6-Jun	**14**	**9**	8	1	1		
11-Jun	**4**	**15**	13	2	1		
13-Jun	**15**	**20**	19	1	1		
14-Jun	**15**	**23**	21	2	1		
15-Jun	**14**	**22**	21	1	1	1	
16-Jun	**14**	**22**	21	1	1		
18-Jun	**15**	**23**	21	2	1	1	
20-Jun	**15**	**23**	21	2	1	1	1
21-Jun	**13**	**23**	21	2	1	1	1
23-Jun	**12**	**23**	21	2	1	1	1
27-Jun	**15**	**20**	19	1	1	1	
28-Jun	**15**	**21**	20	1	1		
4-Jul	**10**	**15**	14	1	1	1	
5-Jul	**11**	**21**	19	2	1	1	
6-Jul	**11**	**18**	17	1	1		
7-Jul	**11**	**18**	17	1	1	1	
8-Jul	**13**	**11**	10	1			
9-Jul	**14**	**13**	12	1			
10-Jul	**13**	**20**	18	2	1	1	
**total**	**244**	**(max 23)**					
