# Supplementary figures and images for: Behavioural synchronization in a multilevel society of feral horses
Source: PLoS One. 2021 Oct 26;16(10):e0258944. doi: 10.1371/journal.pone.0258944 (PMC8547633; doi:10.1371/journal.pone.0258944)

(a)

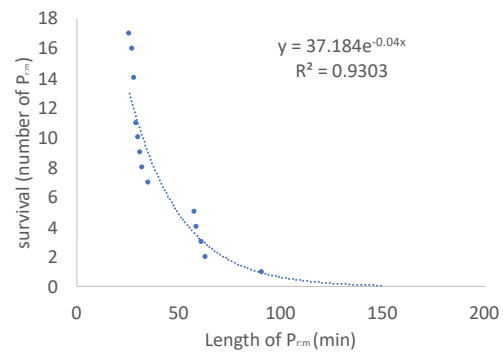

(b)

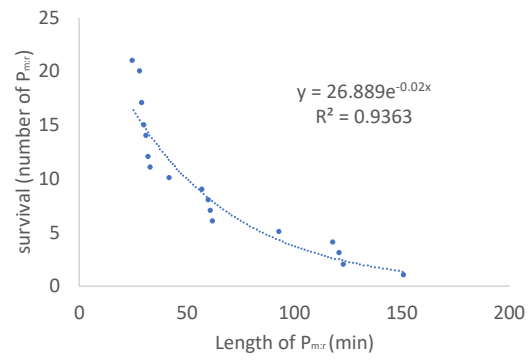

Supplement: S2 Fig — Survival analysis to obtain (a) ΔT01,m and (b) ΔT01,r. The data was fitted to exponential curve. The absolute value of the exponent is considered as the inverse of ΔT01,s (minutes). (PDF) [file pone.0258944.s006.pdf]

(a)

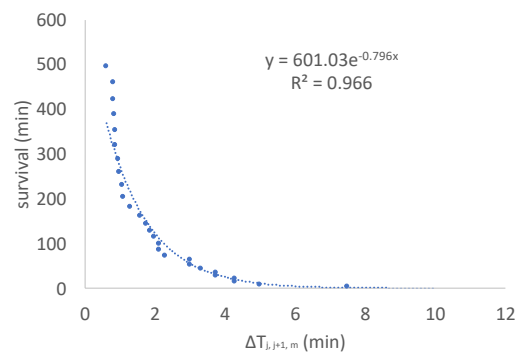

(b)

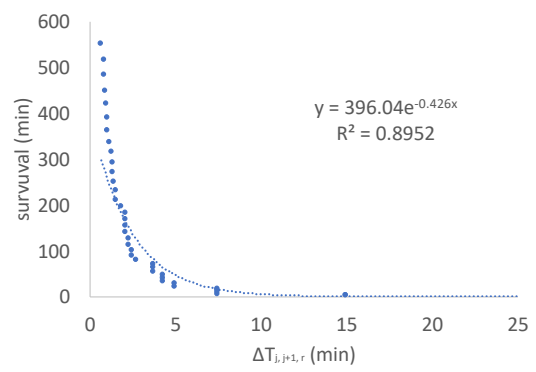

Supplement: S3 Fig — Survival analysis to obtain mimetic coefficient C for (a) Pr:m and (b) Pm:r. The data was fitted to exponential curve. The absolute value of the exponent is considered as C, which is the inverse of ΔTj,j+1,s (minutes). (PDF) [file pone.0258944.s007.pdf]
