# Supplementary material for: Behavioural synchronization in a multilevel society of feral horses
Source: PLoS One. 2021 Oct 26;16(10):e0258944. doi: 10.1371/journal.pone.0258944 (PMC8547633; doi:10.1371/journal.pone.0258944)
Supplement: Peer review history

# Editor report: Decision Letter 0

**DOI:** 10.1371/journal.pone.0258944.r001

**Authors:** Johann Mourier

11 Oct 2021

Behavioural synchronization in a multilevel society of feral horses

PONE-D-21-30005

Dear Dr. Maeda,

We’re pleased to inform you that your manuscript has been judged scientifically suitable for publication and will be formally accepted for publication once it meets all outstanding technical requirements.

Within one week, you’ll receive an e-mail detailing the required amendments. When these have been addressed, you’ll receive a formal acceptance letter and your manuscript will be scheduled for publication.

An invoice for payment will follow shortly after the formal acceptance. To ensure an efficient process, please log into Editorial Manager at http://www.editorialmanager.com/pone/, click the 'Update My Information' link at the top of the page, and double check that your user information is up-to-date. If you have any billing related questions, please contact our Author Billing department directly at authorbilling@plos.org.

If your institution or institutions have a press office, please notify them about your upcoming paper to help maximize its impact. If they’ll be preparing press materials, please inform our press team as soon as possible -- no later than 48 hours after receiving the formal acceptance. Your manuscript will remain under strict press embargo until 2 pm Eastern Time on the date of publication. For more information, please contact onepress@plos.org.

Kind regards,

Johann Mourier, Ph.D.

Academic Editor

PLOS ONE

Journal Requirements:

1. We note that the grant information you provided in the ‘Funding Information’ and ‘Financial Disclosure’ sections do not match. 

When you resubmit, please ensure that you provide the correct grant numbers for the awards you received for your study in the ‘Funding Information’ section.

2. Please include captions for your Supporting Information files at the end of your manuscript, and update any in-text citations to match accordingly. Please see our Supporting Information guidelines for more information: http://journals.plos.org/plosone/s/supporting-information. 

Additional Editor Comments:

Based on my careful reading of the peer-review history and recommendation by Peer community in Network Science, I think that the work is robust and of great interest for the readers of PLOS ONE.

Thus, I am happy to accept this paper for publication in PLOS ONE.

Kind regards

Johann

Reviewers' comments:

# Editor report: Acceptance letter

**DOI:** 10.1371/journal.pone.0258944.r002

**Authors:** Johann Mourier

14 Oct 2021

PONE-D-21-30005 

Behavioural synchronization in a multilevel society of feral horses 

Dear Dr. Maeda:

I'm pleased to inform you that your manuscript has been deemed suitable for publication in PLOS ONE. Congratulations! Your manuscript is now with our production department. 

If your institution or institutions have a press office, please let them know about your upcoming paper now to help maximize its impact. If they'll be preparing press materials, please inform our press team within the next 48 hours. Your manuscript will remain under strict press embargo until 2 pm Eastern Time on the date of publication. For more information please contact onepress@plos.org.

If we can help with anything else, please email us at plosone@plos.org. 

Thank you for submitting your work to PLOS ONE and supporting open access. 

Kind regards, 

PLOS ONE Editorial Office Staff

on behalf of

Dr. Johann Mourier 

Academic Editor

PLOS ONE
